# Improving Protein Crystal Quality by the Without-Oil Microbatch Method: Crystallization and Preliminary X-ray Diffraction Analysis of Glutathione Synthetase from *Pseudoalteromonas haloplanktis*

**DOI:** 10.3390/ijms12096312

**Published:** 2011-09-23

**Authors:** Antonello Merlino, Irene Russo Krauss, Antonella Albino, Andrea Pica, Alessandro Vergara, Mariorosario Masullo, Emmanuele De Vendittis, Filomena Sica

**Affiliations:** 1 Dipartimento di Chimica “Paolo Corradini”, Università di Napoli Federico II, Complesso Universitario di Monte Sant’Angelo, Via Cinthia, Naples I-80126, Italy; E-Mails: antonello.merlino@unina.it (A.M.); irene.russokrauss@unina.it (I.R.K.); andrea.pica@unina.it (A.P.); alessandro.vergara@unina.it (A.V.); 2 Istituto di Biostrutture e Bioimmagini, CNR, Via Mezzocannone 16, Naples I-80134, Italy; 3 Dipartimento di Biochimica e Biotecnologie Mediche, Università di Napoli Federico II, Via Pansini 5, Naples I-80131, Italy; E-Mails: antonella.albino@unina.it (A.A.); mario.masullo@uniparthenope.it (M.M.); devendittis@dbbm.unina.it (E.V.); 4 Dipartimento di Studi delle Istituzioni e dei Sistemi Territoriali, Università di Napoli “Parthenope”, Via Medina 40, Naples I-80133, Italy

**Keywords:** crystal quality, without-oil microbatch, glutathione synthetase, psychrophile, X-ray crystallography

## Abstract

Glutathione synthetases catalyze the ATP-dependent synthesis of glutathione from l-γ-glutamyl- l-cysteine and glycine. Although these enzymes have been sequenced and characterized from a variety of biological sources, their exact catalytic mechanism is not fully understood and nothing is known about their adaptation at extremophilic environments. Glutathione synthetase from the Antarctic eubacterium *Pseudoalteromonas haloplanktis* (*Ph*GshB) has been expressed, purified and successfully crystallized. An overall improvement of the crystal quality has been obtained by adapting the crystal growth conditions found with vapor diffusion experiments to the without-oil microbatch method. The best crystals of *Ph*GshB diffract to 2.34 Å resolution and belong to space group *P*2_1_2_1_2_1_*,* with unit-cell parameters *a* = 83.28 Å, *b* = 119.88 Å, *c* = 159.82 Å. Refinement of the model, obtained using phases derived from the structure of the same enzyme from *Escherichia coli* by molecular replacement, is in progress. The structural determination will provide the first structural characterization of a psychrophilic glutathione synthetase reported to date.

## 1. Introduction

The tripeptide glutathione (GSH) is the most abundant antioxidant molecule in cells and has multiple biological functions. Besides its protective role in counteracting oxidative free radical species and in the removal of toxic metals, GSH is involved in redox homeostasis, amino acid transport and metabolism of therapeutic drugs, mutagenesis and carcinogenesis; in addition, GSH is also involved in cell cycle regulation, cell signaling, and apoptosis [1–3]. The synthesis of GSH from its constituent amino acids l-glutamic acid, l-cysteine and glycine involves two ATP-requiring enzymatic steps catalyzed by glutamate cysteine ligase (GshA) and glutathione synthetase (GshB), respectively. In particular, glutathione synthetase catalyzes the synthesis of GSH from the γ-l-glutamyl-l-cysteine and glycine in the presence of ATP and magnesium ion [4]. This process involves the formation of an acyl phosphate on the cysteinyl moiety in l-γ-glutamyl-l-cysteine, followed by the attack of the glycine and formation of an enzyme-product complex, which finally dissociates with the release of GSH, ADP and phosphate (1).

(1) L-γ-glutamyl-L-cysteine+glycine+ATP→GSH+ADP+Pi

Glutathione synthetase has been purified, sequenced and characterized from different sources, including yeast [5], human [6,7], rat [8] and *Escherichia coli* [9]. The comparison of human enzyme with other eukaryotic proteins reveals a high sequence variability, with identity ranging from 18% to 69% [10]. To date, glutathione biosynthesis has never been studied in a psychrophilic source, even though it is known that some antioxidant proteins are covalently modified and likely regulated by cellular thiols (see for example [11–13]).

*Pseudoalteromonas haloplanktis*, a psychrophilic Gram-negative bacterium collected from Antarctic seawater (growth temperature interval, 4–20 °C), produces a glutathione synthetase (*Ph*GshB), whose polypeptide chain is made up of 315 residues, corresponding to a molecular mass of 36 kDa. Sequence alignments show a significant similarity of *Ph*GshB with its mesophilic counterpart from *E. coli* (69% sequence identity), whose structure has been already determined [14,15]. With the aim of gaining insights on the mechanism of cold adaptation of glutathione synthetases and to shed light on the exact mechanism of catalytic action of these enzymes, we have undertaken the structural and functional characterization of *Ph*GshB. In this article, we describe the expression, purification, crystallization and preliminary X-ray diffraction studies of the protein. We found that the adaptation of crystallization conditions found using vapor diffusion experiments to a modified microbatch method significantly improves the size and the diffracting power of *Ph*GshB crystals.

## 2. Results and Discussion

### 2.1. Crystallization of PhGshB

**Figure 1:**
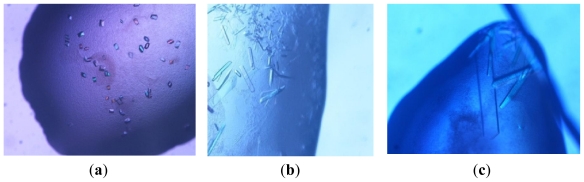
Image of typical cubic (**a**) and rod-like (**b**) r*Ph*GshB crystals grown by vapor diffusion; (**c**) crystals grown by without-oil microbatch technique.

A recombinant form of *Ph*GshB (r*Ph*GshB) with the C-terminal lysine replaced by the extrapeptide Leu-Glu-His_6_-tag, LE(H)_6_, has been successfully expressed, purified and crystallized using vapor-diffusion and small-scale batch (microbatch) methods. The purified r*Ph*GshB showed a single band of approximately 36 kDa on SDS-PAGE, which is in good agreement with the theoretical molecular mass predicted by the amino acid sequence. Initial screenings using commercially available solutions (Crystal Screen kits I and II, and Index kit from Hampton Research, Laguna Niguel, USA, www.hamptonreaserch.com) revealed several promising conditions for the crystallization of r*Ph*GshB. All favorable conditions were characterized by the presence of polyethylene glycol as precipitating agent. In particular, cubic and rod-like crystals appeared within 2–5 days using a 20 mg mL^−1^ protein concentration in the hanging-drop method from crystallization conditions with the reservoir solution containing 30% w/v MPEG 2K, 0.1 M potassium thiocyanate, and 25% w/v PEG 3350, 0.1 M HEPES pH 7.5, respectively. At this stage, maximum size of cubic crystals (Figure 1a) was 0.05 mm × 0.05 mm × 0.04 mm, whereas that for rod-like crystals was 0.1 mm × 0.1 mm × 0.2 mm. The quality of the crystals was improved by fine-tuning the concentration of protein (10.0–30.0 mg mL^−1^) and precipitants and evaluating the effect of several salts, like ammonium sulfate, sodium malonate, sodium potassium tartrate and tacsimate. The best crystals (rod-like, Figure 1b) were obtained from a crystallization solution containing 10% w/v PEG 20K, 5% v/v tacsimate and 0.1 M HEPES pH 7.5 and r*Ph*GshB at 20.0 mg mL^−1^.

Preliminary X-ray diffraction data showed that cubic crystals are intrinsically disordered and that the largest rod-like crystals obtained by vapor diffusion diffract at most at 3.5 Å resolution, they belong to the space group *P*2_1_2_1_2_1_*,* with unit-cell parameters *a* = 82.81 Å, *b* = 119.94 Å, *c* = 159.32 Å. Further optimization of the crystallization conditions to grow larger and thicker crystals suitable for diffraction data collection at high resolution were performed using the microbatch method. This procedure consists of slowly and thoroughly mixing the precipitant with the protein and then placing the mixture in a well-sealed container. Usually the drop is incubated under silicon oil to prevent too rapid dehydration [16]. The benefits of microbatch have been well documented [16,17]. In the present case, a modified microbatch method, that we define without-oil microbatch, has been used: the drop is stored in the presence of a reservoir with the same precipitant concentration, to avoid drop evaporation [18]. A similar microbatch technique, which does not utilize oil (Small-Scale Batch without Oil) has been developed at the University of Wisconsin [19], and has been successfully used for many years, though no claim is made for its originality. As reported in other cases [18,19], we found that crystallization occurred using 60%–80% of the concentration of the precipitant required in the hanging drop experiment. The best crystals of r*Ph*GshB (maximum size of 0.2 mm × 0.3 mm × 0.4 mm) were obtained by mixing a solution containing 14% w/v PEG 20K, 0.2 M HEPES pH 7.5, 10% v/v tacsimate with an equal volume of protein solution at 40 mg mL^−1^ (Figure 1c). These crystals diffract to 2.34 Å resolution and are isomorphous to those obtained by hanging drop experiments, with unit-cell parameters *a* = 83.28 Å, *b* = 119.88 Å, *c* = 159.82 Å (Table 1). Matthews coefficient calculations suggested the presence of four chains of r*Ph*GshB (*V*_M_ = 2.79 Å^3^ Da^−1^, 56% solvent content [20]) in the asymmetric unit. The application of the molecular replacement, as detailed in the Experimental Section, allowed the identification of orientation and position of the four chains in the asymmetric unit that gave a satisfactory fit of the experimental data. Rebuilding and refinement of the whole structure is in progress. Calculated preliminary (*F*_o_-*F*_c_) and (2*F*_o_-*F*_c_) difference Fourier maps are of excellent quality.

## 3. Experimental Section

### 3.1. Expression and Purification of rPhGshB

The putative gene encoding GshB in *P. haloplanktis* (*Ph*gshB; ID 3708001) [21] was amplified by PCR using specific oligonucleotides allowing its cloning in the pET-28a(+) vector. The forward and reverse primers used were 5′d–A_–12_AGGCACAGCCC•*ATG*•GCA•ATT_9_–3′ and 5′d–A_960_AC•GCT•AAC•CTC•GAG•AGC•GAG•TCG•T_936_–3′, respectively. Numbering in primers begins from starting codon (italics), whereas underlined letters indicate mismatches introduced to create the *Nco*I and *Xho*I restriction sites. The amplified segment was digested with *Nco*I and *Xho*I and cloned in pET-28a(+) previously digested with the same endonucleases. The new construct was controlled by nucleotide sequencing and used to transform the *E. coli* BL21(DE3) strain. A culture of this transformant was grown at 37 °C up to 0.6 OD_600_ and the heterologous expression was induced for 2 h upon the addition of 0.1 mg mL^−1^ isopropyl-β-thiogalactopyranoside. Bacterial cells were collected by centrifugation, resuspended in 20 mM Tris-HCl buffer pH 7.8, and then lysed by a French Press (Aminco, USA) to obtain a cell homogenate. This sample was then centrifuged at 30,000 × g for 1 h and the supernatant was used as starting material for the purification by affinity chromatography on Ni-NTA agarose of r*Ph*GshB, which had the *C*-terminal lysine replaced by the extrapeptide LE(H)_6_. To this aim, the supernatant was added in batch to the Ni-NTA Agarose resin, equilibrated in 20 mM Tris-HCl buffer pH 7.8. After incubation overnight at 4 °C, the slurry was poured in a column, which was extensively washed with the same buffer supplemented with 10 mM imidazole-HCl. The bound r*Ph*GshB was then eluted with 20 mM Tris-HCl buffer pH 7.8 supplemented with 50 mM imidazole-HCl and pure protein fractions, as analysed by SDS-polyacrylamide gel elecrophoresis, were pooled together, concentrated by ultrafiltration, and stored at −20 °C in 20 mM Tris-HCl buffer pH 7.8 supplemented with 50% (v/v) glycerol. Nearly 20 mg of pure r*Ph*GshB were obtained from a 1 L culture of the transformant.

### 3.2. Crystallization of rPhGshB

Purified r*Ph*GshB was concentrated to 40 mg mL^−1^ in 20 mM Tris-HCl pH 7.8. Crystallization was performed at 20 °C by the hanging-drop vapor-diffusion method with 0.2 μL of protein and 0.2 μL of reservoir. The following commercially available screens were used: Crystal Screen kits I and II, and Index kit from Hampton Research.

Optimization of the crystallization conditions was performed by fine-tuning the protein and precipitant concentrations using a drop consisting of 0.5 μL protein solution and 0.5 μL precipitant solution and a reservoir volume of 500 μL.

Cubic crystals were obtained within 2–5 days from drops containing r*Ph*GshB (20 mg mL^−1^ in 10 mM Tris-HCl pH 7.8) 30% w/v MPEG 2K and 0.1 M potassium thiocyanate, whereas rod-like crystals were obtained using the same protein solution and a precipitant solution containing 25% w/v PEG 3350 and 0.1 M HEPES pH 7.5. An improvement in the quality of crystals was obtained using different salts and precipitant agents. In particular, well-shaped crystals were grown using 10% w/v PEG 20K, 5% v/v tacsimate, 0.1 M HEPES pH 7.5 as a precipitant solution. However, these crystals only diffract at 3.5 Å resolution. A further improvement was obtained adopting the without-oil microbatch method. In this technique, a solution of 40 mg mL^−1^ protein in 20 mM Tris-HCl pH 7.8 was directly mixed with an equal amount of precipitating solution (final concentration: 7% w/v PEG20K, 5% v/v tacsimate, 0.1 M HEPES pH 7.5). In all the experiments, standard 24-well linbro plates (Hampton Research, Laguna Niguel, USA, www.hamptonreaserch.com) have been used.

### 3.3. Data collection and Processing

Preliminary diffraction data (3.5 Å resolution) on the rod-like crystals obtained by vapor diffusion were collected at the Institute of Biostructures and Bioimages (Naples, Italy), at 100 K using a Rigaku MicroMax-007 HF generator producing Cu *K*α radiation and equipped with a Saturn944 CCD detector.

Higher resolution data (2.34 Å) were collected at 100 K on the crystals obtained by without-oil microbatch at the synchrotron beamline XRD1 of Elettra (Trieste, Italy). Cryoprotection of the crystals was achieved by rapid soaking (1–2 s) in a solution consisting of 12% w/v PEG 20K, 0.1 M HEPES buffer pH 7.5, 5% v/v tacsimate, 30% v/v glycerol. An oscillation range of 0.5° and an X-ray dose corresponding to about 5 s exposure were adopted for all experiments. The data sets were indexed, processed and scaled using the *HKL*-2000 program package (Table 1) [22]. The precision-indicating merging *R*-factors (*R*_pim_ and *R*_rim_) were calculated using the program RMERGE [23,24].

### 3.4. Structure Determination

The structure of the enzyme was solved by molecular replacement techniques using the program Phaser [25] and the single chain structure of GshB from *E. coli* as search model (PDB code 1GSA [14]). To avoid bias, ligands and water molecules were removed from the model prior to structure factors and phases calculation. The solution had an *R*-factor of 0.39.

## 4. Conclusions

In the current study, the glutathione synthetase from the Antarctic eubacterium *Pseudoalteromonas haloplanktis* was crystallized, X-ray diffraction data collected and the phase problem solved.

An overall improvement of the crystal quality was obtained by adapting the crystal growth conditions found with vapor diffusion experiments to the without-oil microbatch method. In general, the more stable the system in the crystallization process, the higher is the quality of the crystals. These conditions are better realized in the microbatch method, where the crystallization components are mixed to their final concentration at the beginning of the experiment. A significant drawback of the under-oil microbatch method is the slow evaporation of water from the crystallization drops resulting sometimes in the formation of salt deposits that interfere with protein crystal growth. Our results, together with recent literature data [18,19], suggest that the adaptation of crystallization conditions to without-oil microbatch method could be a general strategy to convert poorly diffracting crystals into diffraction-quality ones, but further studies are needed to test the validity of this hypothesis.

## Figures and Tables

**Table 1:** X-ray diffraction data collection statistics.

	r*Ph*GshB
Space group	*P*2_1_2_1_2_1_
**Cell parameters:**	
*a* (Å)	83.28
*b* (Å)	119.88
*c* (Å)	159.82
Resolution limits (Å)	50.00–2.34
Highest resolution shell (Å)	2.45–2.34
No. of observations	337677
No. of unique reflections	66744
Completeness (%)	97.1 (85.0)
I/σ (I)	20.5 (2.6)
Average multiplicity	5.1 (2.5)
*R*_merge_ (%)	11.5 (40.5)
*R*_pim_	5.0 (27.5)
*R*_rim_	12.7 (49.6)
Mosaicity	0.3

Note: Values in parentheses correspond to the highest resolution shells.

## References

[1] Dalle-Donne I, Rossi R, Giustarini D, Colombo R, Milzani A (2007). S-glutathionylation in protein redox regulation. Free Radic Biol Med.

[2] Circu ML, Aw TY (2008). Glutathione and apoptosis. Free Radic Res.

[3] Pallardo FV, Markovic J, Garcia JL, Vina J (2009). Role of nuclear glutathione as a key regulator of cell proliferation. Mol Asp Med.

[4] Yamaguchi H, Kato H, Hata Y, Nishioka T, Kimura A, Oda J, Katsube Y (1993). Three-dimensional structure of the glutathione synthetase from *Escherichia coli* B at 2.0 Å resolution. J Mol Biol.

[5] Mooz ED, Meister A (1967). Tripeptide (glutathione) synthetase: Purification, properties and mechanism of action. Biochemistry.

[6] Gali RR, Board PG (1995). Sequencing and expression of a cDNA for human glutathione synthetase. Biochem J.

[7] Ota T, Suzuki Y, Nishikawa T, Otsuki T, Sugiyama T, Irie R, Wakamatsu A, Hayashi K, Sato H, Nagai K (2004). Complete sequencing and characterization of 21,243 full-length human cDNAs. Nat Genet.

[8] Huang CS, He W, Meister A, Anderson ME (1995). Amino acid sequence of rat kidney glutathione synthetase. Proc Natl Acad Sci USA.

[9] Gushima H, Yasuda S, Soeda E, Yokota M, Kimura A (1984). Complete nucleotide sequence of the *E. coli* glutathione synthetase gsh-II. Nucleic Acids Res.

[10] Polekhina G, Board PG, Gali RR, Rossjohn J, Parker MW (1999). Molecular basis of glutathione synthetase deficiency and a rare gene permutation event. EMBO J.

[11] Castellano I, Di Maro A, Ruocco MR, Chambery A, Parente A, Di Martino MT, Parlato G, Masullo M, De Vendittis E (2006). Psychrophilic superoxide dismutase from *Pseudoalteromonas haloplanktis*: Biochemical characterization and identification of a highly reactive cysteine residue. Biochimie.

[12] Castellano I, Ruocco MR, Cecere F, Di Maro A, Chambery A, Michniewicz A, Parlato G, Masullo M, De Vendittis E (2008). Glutathionylation of the iron superoxide dismutase from the psychrophilic eubacterium *Pseudoalteromonas haloplanktis*. Biochim Biophys Acta Protein Proteonomics.

[13] Merlino A, Russo Krauss I, Castellano I, De Vendittis E, Rossi B, Conte M, Vergara A, Sica F (2010). Structure and flexibility in cold-adapted iron superoxide dismutases: The case of the enzyme isolated from *Pseudoalteromonas haloplanktis*. J Struct Biol.

[14] Matsuda K, Mizuguchi K, Nishioka T, Kato H, Go N, Oda J (1996). Crystal structure of glutathione synthetase at optimal pH: Domain architecture and structural similarity with other proteins. Protein Eng.

[15] Hara T, Kato H, Katsube Y, Oda J (1996). A pseudo-Michaelis quaternary complex in the reverse reaction of a ligase: Structure of *Escherichia coli* B glutathione synthetase complexed with ADP, glutathione, and sulfate at 2.0 Å resolution. Biochemistry.

[16] Chayen NE, Shaw Stewart PD, Maeder DL, Blow DM (1990). An automated system for micro-batch protein crystallization and screening. J Appl Cryst.

[17] Chayen NE (1999). Recent advances in methodology for the crystallization of biological macromolecules. J Cryst Growth.

[18] Martins PM, Pessoa J, Sarkany Z, Rocha F, Damas AM (2008). Rationalizing protein crystallization Screenings through water equilibration theory and protein solubility data. Cryst Growth Des.

[19] Rayment I (2002). Small-scale batch crystallization of proteins revisited: An underutilized way to grow large protein crystals. Structure.

[20] Matthews BW (1968). Solvent content of protein crystals. J Mol Biol.

[21] Medigue C, Krin E, Pascal G, Barbe V, Bernsel A, Bertin PN, Cheung F, Cruveiller S, D’Amico S, Duilio A (2005). Coping with cold: The genome of the versatile marine Antarctica bacterium *Pseudoalteromonas haloplanktis* TAC125. Genome Res.

[22] Otwinowski Z, Minor W (1997). Processing of X-ray diffraction data collected in oscillation mode. Methods Enzymol.

[23] Evans P (2006). Scaling and assessment of data quality. Acta Cryst D.

[24] Weiss MS (2001). Global indicators of X-ray data quality. J Appl Crystallogr.

[25] Storoni LC, McCoy AJ, Read RJ (2004). Likelihood-enhanced fast rotation functions. Acta Crystallogr D Biol Crystallogr.

